# Investigating the Biological Characteristics and Pathogenic Potential of *Listeria innocua* Isolated from Food Through Comparative Genomics

**DOI:** 10.3390/microorganisms13112525

**Published:** 2025-11-02

**Authors:** Bo Zhang, Runlai Cao, Qilin Wang, Pan Hu, Yacong Li, Ziyu Liu, Zhuqing Xue, Weiyang Wang, Shasha Zhang, Xiaoxu Wang

**Affiliations:** 1 Key Laboratory of Special Animal Epidemic Disease, Ministry of Agriculture and Rural Affairs, Jilin Provincial International Cooperation Key Laboratory for Science and Technology Innovation of Special Animal and Plants, Institute of Special Animal and Plant Sciences, Chinese Academy of Agricultural Sciences, Changchun 130112, China; zbjlu97@163.com (B.Z.); caorunlai@caas.cn (R.C.); wangqilin@caas.cn (Q.W.); lycong910@163.com (Y.L.); 13844790017@163.com (Z.L.); xzqblblbl@163.com (Z.X.); weiyangwang8779@163.com (W.W.); 2 State Key Laboratory for Diagnosis and Treatment of Severe Zoonotic Infectious Diseases, Key Laboratory for Zoonosis Research of the Ministry of Education, Institute of Zoonosis, and College of Veterinary Medicine, Jilin University, Changchun 130062, China; hupan84@163.com; 3 Panjin Center for Inspection and Testing, Panjin 124010, China; hbgcyzr@163.com

**Keywords:** comparative genomics, *Listeria innocua*, pan-genomics, pathogenic potential, virulence, biodiversity

## Abstract

*L. monocytogenes* is a common foodborne pathogen that typically causes infections through the consumption of food contaminated with this bacterium. This study seeks to elucidate the biodiversity as well as evolutionary characteristics of *L. innocua* strains from different regions using comparative genomics, exploring the virulence and pathogenic potential of these strains. The findings are expected to deepen our understanding of *L. innocua* and provide valuable reference for public health risk assessment related to this bacterium. We performed comparative genomics on 108 food-source *L. innocua* isolates sourced from the USA, England, China, and Egypt to explore their biological traits and assess their pathogenic potential by predicting virulence and antibiotic resistance genes, with subsequent validation of pathogenicity through animal studies. Pan-genomic analysis showed that geographically distinct *L. innocua* strains possess open genomes, offering a stable genetic basis that facilitates adaptation to diverse environments. Through virulence gene prediction, we found that *L. innocua* strains from different regions harbor virulence genes identical to those found in pathogenic *L. monocytogenes*, such as *inlA* and *inlB*, as well as internal genes that may enhance the pathogenic potential of the strains. This finding demonstrates that *L. innocua* strains exhibit pathogenic potential. To validate their virulence, we subsequently conducted virulence assays utilizing the Galleria mellonella larval model. Following infection with *L. innocua*, 100% mortality was observed in a subset of Galleria mellonella larvae, albeit with a delayed time to death compared to *L. monocytogenes* infection. This indicates that while *L. innocua* exhibits attenuated virulence relative to *L. monocytogenes*, it retains pathogenicity. Consequently, the potential contribution of *L. innocua* to listeriosis cannot be overlooked in public health risk assessments. *L. innocua* strains isolated from food can carry virulence and resistance genes identical to those found in pathogenic *L. monocytogenes* strains, indicating that these *L. innocua* strains possess certain virulence and pathogenic potential, which was further validated through subsequent animal experimentation. This study enhances our genomic understanding of *L. innocua* and underscores that detecting its key virulence genes is critical for public health safety, thereby providing valuable insights into its pathogenic potential.

## 1. Introduction

*Listeria* is a facultatively anaerobic, Gram-positive rod-shaped bacterium. Currently, the prokaryotic nomenclature database LPSN contains 30 registered species of *Listeria* (last accessed on 1 August 2025) [1]. Among these spp., *L. monocytogenes* and *L. ivanovii* are typically regarded as pathogenic strains. However, the widespread environmental prevalence of *L. innocua* necessitates consideration of its potential impact, despite the primary public health concern being attributed to *L. monocytogenes* [2,3].

Typically, *L. monocytogenes* infections occur through foodborne transmission, meaning the infection is caused by the consumption of contaminated food [4,5]. During the infection process, virulence genes play a crucial role [6]. The invasion of host cells by *L. monocytogenes* is initiated by the inlA and inlB proteins, which mediate bacterial attachment to the host’s E-Cadherin and Met receptors, respectively. This interaction triggers receptor-mediated endocytosis, leading to bacterial internalization within a vacuole. To escape this vacuolar compartment, the bacterium expresses the virulence factors *plcA*, *plcB*, and *hly*. Subsequently, the actA protein orchestrates actin polymerization, generating the propulsive force that enables cell-to-cell spread [7,8]. Recent studies, including one by Moura et al. [9], have demonstrated that *L. innocua* can cross the intestinal epithelium and disseminate systemically to organs such as the liver and spleen. Despite this invasive capability, its virulence remains considerably lower than that of *L. monocytogenes*. Genetic analyses by Moura et al. [9] and Rossi et al. [10] collectively suggest that food-borne *L. innocua* strains may act as reservoirs for pathogenic listeriosis strains. Notably, these studies identified that key *L. monocytogenes* virulence determinants, such as *inlA* and *hly*, can be present not only in atypical *L. innocua* but also in other species like *L. welshimeri* and *L. seeligeri*. Mafuna et al. [11] have observed that the virulence genes of *L. monocytogenes* may also be carried by *L. innocua* strains. Matto et al. [12]. found that *L. innocua* was responsible for a proportion of bovine listeriosis cases investigated in Uruguay. Therefore, investigating the presence of virulence genes in *L. innocua* strains from different regions will aid in further understanding the potential pathogenicity of *L. innocua* strains.

Comparative genomic analysis effectively identifies regional variations among *L. innocua* strains and their differences from *L. monocytogenes*. This enhances our understanding of the biological characteristics of *L. innocua*, reveals variations in its virulence gene repertoire, and thus addresses a critical knowledge gap regarding its potential pathogenicity. Risk assessment offers a scientific foundation for updating food safety standards. The pathogenic potential of *L. innocua* highlights the need to incorporate this species into listeriosis risk models, thereby improving their accuracy and comprehensiveness.

The advent and proliferation of next- and third-generation sequencing have led to an increasing volume of *L. innocua* genomic sequences being deposited in public databases [13,14]. Comparative genomics analysis of *L. innocua* strains from distinct regions enables the enhancement of our comprehension of the genetic mechanisms underlying the adaptation of *L. innocua* to various environments and its potential virulence [15,16]. This study seeks to elucidate the biological diversity and evolutionary traits of *L. innocua* strains from distinct regions using comparative genomics, exploring the virulence and pathogenic potential of these strains.

## 2. Materials and Methods

### 2.1. Data Retrieval and Management

In this study, we retrieved and downloaded all *L. innocua* strains isolated from food sources from the NCBI Genome Database (last accessed 24 February 2025), comprising a total of 108 whole-genome sequences. This dataset includes 96 *L. innocua* strains from four different regions (18 strains from the USA, 55 strains from England, 3 strains from China, and 20 strains from Egypt). Additionally, 12 *L. innocua* strains isolated from food in our laboratory were included, adding to the dataset for the China region. Isolated strains were included based on the following criteria: a genome size between 2.8 and 3.2 Mb, a GC content of 37–38%, ≤20 contigs, and no identical matches in public databases upon comparative analysis. For this study, we retrieved all publicly available genomes of food-derived *L. innocua* from the NCBI database and combining them with the whole-genome sequences of *L. innocua* strains isolated from food in our laboratory for subsequent analysis. Comprehensive genomic features of these strains are provided in Tables S1 and S2.

### 2.2. Pan-Genomic Analysis of L. innocua from Different Geographic Origins

To investigate these common characteristics of *L. innocua* strains from diverse geographical regions, we conducted a pan-genome analysis to define their core gene set. In brief, we annotated all genomic sequences with Prokka (v1.14.6) [17] using its default parameters. We then conducted a pan-genome analysis of the resulting annotations using Roary (v3.11.2) [18] with its default settings. The parameters for defining the core genome of each isolate (99% cutoff) and for BLASTP-based clustering (85% identity cutoff) were determined through sensitivity analysis [19,20].

The pan-genome was classified into four categories based on its distribution across isolates: the core genome (100% of isolates), the soft-core genome (≥95% of isolates), the shell or accessory genome (15–95% of isolates), and the cloud or unique genome (<15% of isolates) [21].

### 2.3. Multilocus Sequence Typing (MLST) Analysis

MLST of *L. innocua* was performed targeting seven housekeeping genes (*abcZ*, *bglA*, *cat*, *dapE*, *dat*, *ldh*, *lhkA*) as described [22]. The corresponding MLST profiles were obtained from the *Listeria* database at the Pasteur Institute, France. Sequence types (STs) and clonal complex (CC) assignments for each genome were subsequently determined by aligning sequencing reads to these reference profiles using MLST software (v.2.18.0) [21].

### 2.4. Phylogenetic Analysis

To examine the phylogenetic relationships among the 108 geographically distinct *L. innocua* isolates, we first extracted and aligned all core single-copy genes utilizing MAFFT (v7.490). The resulting alignments were concatenated for each strain in a consistent gene order, and poorly aligned regions were trimmed with GBLOCKS 0.91b. A maximum likelihood (ML) tree was subsequently constructed with MEGA 11. The robustness of the phylogenetic tree was assessed by employing different tree-building methods. Final visualization and annotation with geographic origin and ST were performed using the Interactive Tree of Life (iTOL, v6) online tool, applying midpoint rooting [1].

### 2.5. Functional Features of Core Genes

To characterize the functional attributes of the core genes in geographically diverse *L. innocua* strains, we conducted enrichment analyses against the Gene Ontology (GO) and Kyoto Encyclopedia of Genes and Genomes (KEGG) [23], and results were then integrated.

### 2.6. Prediction of Virulence Factors Genes and Antibiotic Resistance Genes of L. innocua

The existence of virulence and antibiotic resistance genes in the *L. innocua* genomes was determined by predictive genomic analysis, leveraging the Virulence Factors of Pathogenic Bacteria (VFDB) and Comprehensive Antibiotic Resistance (CARD) databases [11,24]. The findings were aggregated and visualized in a heatmap.

### 2.7. Prediction of Plasmids of L. innocua

As mobile genetic elements (MGEs), plasmids play a crucial role in microbial evolution by promoting genetic diversity and adaptation [25]. The plasmid database PLSDB was utilized to detect plasmids in the *L. innocua* genome [26]. For the visual representation of the prediction data, a heatmap was constructed.

### 2.8. Prediction of LGIs and SSIs of L. innocua

We predicted *Listeria* Genomic Islands (LGIs) and Stress Survival Islands (SSIs) in *L. innocua* strains to elucidate the distribution of resistance genes and assess their adaptive potential to various environments. The findings were visualized in a heatmap.

### 2.9. Prediction of CRISPR-Cas Systems of L. innocua

The CRISPR-Cas system in *L. innocua* genomes was predicted and typed using CRISPRCasFinder (v1.1.2) [27]. A system was deemed operational only if both CRISPR arrays and associated cas genes were present; these high-confidence systems were retained for subsequent analysis. The final results were compiled and visualized in a heatmap.

### 2.10. Verifying the Pathogenicity of L. innocua Strains

To further verify the potential pathogenicity of *L. innocua*, an in vivo infection model using Galleria mellonella larvae was employed. Briefly, purchased larvae (Tianjin Huivude Biotechnology Co., Ltd., China) were acclimatized for three days prior to experimentation. In the infection model, larvae were randomly assigned to 13 groups (*n* = 10, with one group serving as the control and the remaining 12 groups each assigned a unique *L. innocua* strain). Each larva in the test groups was injected with 10 μL of a bacterial suspension (1 × 10^8^ CFU/mL), whereas control larvae received 10 μL of PBS. The entire experiment was conducted in triplicate. After a 24-h incubation period at 37 °C, mortality rates were statistically analyzed using the Chi-square test [28].

## 3. Results

### 3.1. Genome Statistics and General Features

The detailed characteristics of the analyzed *L. innocua* strains are compiled in Tables S1 and S2. Among them, 18 strains were included from the USA, with a mean genome size of 3.1 (3.0–3.2) Mbp, a mean GC content of 37.1 (37.0–37.5)%, contig count ≤ 26 and a mean N50 of 523.8 Kbp. 55 *L. innocua* isolates were included from England, characterized by a mean genome size of 3.0 (2.8–3.2) Mbp, a mean GC content of 37.4 (37.0–37.5)%, contig count ≤ 29 and a mean N50 of 359.1 Kbp. 15 *L. innocua* isolates were included from China, with a mean genome size of 2.9 (2.8–3.1) Mbp, a mean GC content of 37.4 (37.0–37.5)%, contig count ≤ 10 and a mean N50 of 1442.9 Kbp. 20 *L. innocua* isolates were included from Egypt, showing a mean genome size of 3.0 (2.9–3.1) Mbp, a mean GC content of 37.5%, contig count ≤ 74 and a mean N50 of 131.2 Kbp.

### 3.2. Pan-Genomic Analysis of L. innocua Strains in Distinct Areas

**Figure 1:**
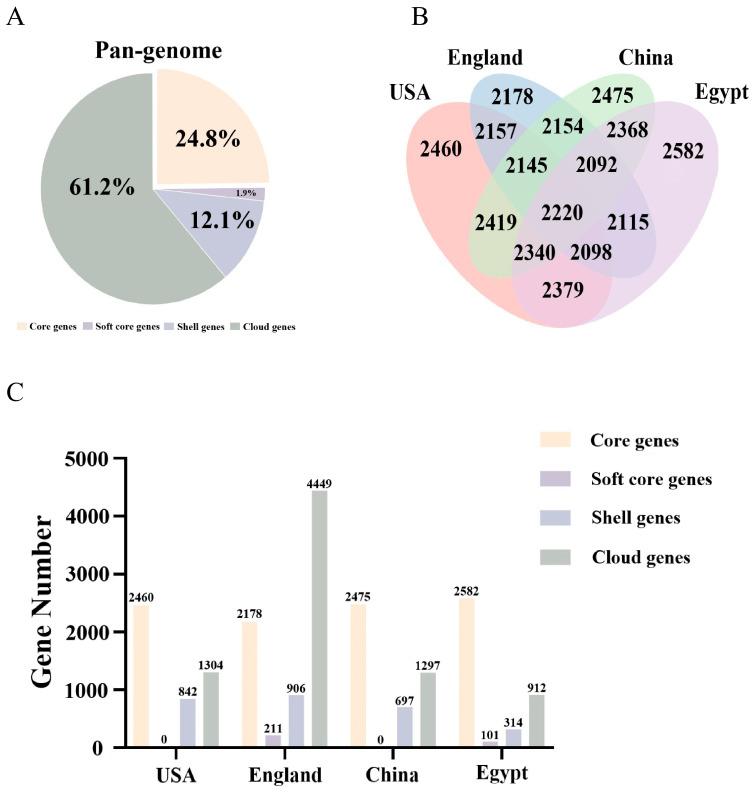
Pan-genome analysis of *L. innocua* from four geographic regions. (**A**) Composition of the pan-genome across regions. (**B**) Venn diagram of the core genes shared between regions. (**C**) Gene count distribution across the core, soft-core, shell, and cloud genomic compartments.

According to the pan-genomic categorization, the gene repertoire of the geographically diverse *L. innocua* strains was composed of 2,220 core genes (24.8%), 169 soft-core genes (1.9%), 1,078 shell genes (12.1%), and 5,473 cloud genes (61.2%), as detailed in Figure 1A. The pan-genomic composition of *L. innocua* varies across different regions. Among the *L. innocua* strains, those the USA harbor 2460 core genes, 0 soft-core genes, 842 accessory genes, and 1304 unique genes. Those from England harbor 2178 core genes, 211 soft-core genes, 906 accessory genes, and 4449 unique genes. Those from China harbor 2475 core genes, 0 soft-core genes, 697 accessory genes, and 1297 unique genes. Those from Egypt harbor 2582 core genes, 101 soft-core genes, 314 accessory genes, and 912 unique genes (Figure 1B,C). Collective analysis of genomic, pan-genomic, and core-genomic features demonstrated that geographically distinct *L. innocua* strains harbor open genomes. This genomic architecture provides a stable genetic foundation that facilitates their adaptation to diverse and heterogeneous environments.

### 3.3. MLST and Phylogenetic Analysis

MLST was applied at the whole-genome level to elucidate the genetic relatedness of *L. innocua* strains from distinct geographical origins, and the phylogenetic relationships were determined among various sequence types (STs). In USA, the most common among *L. innocua* strains was ST1008 (*n* = 15, 83.3%) and CC1008 (*n* = 15, 83.3%) (Table S3). In England, the most common was ST603 (*n* = 8, 14.5%) and CC600 (*n* = 8, 14.5%) (Table S3). In China, the most common was ST474 (*n* = 3, 20%) and CC474 (*n* = 3, 20%) (Table S3). In Egypt, the most common was ST530 (*n* = 20, 100%) and CC530 (*n* = 20, 100%) (Table S3). The MLST predominant types of *L. innocua* strains isolated from food in different regions vary, indicating that the predominant STs of *L. innocua* exhibit regional specificity.

**Figure 2:**
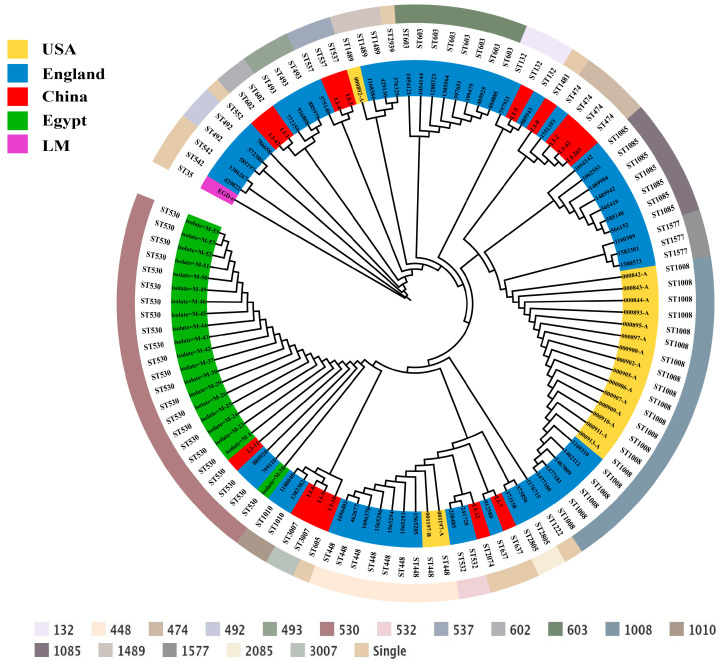
Phylogenetic tree of *L. innocua* strains from distinct geographical areas, annotated with their corresponding STs in the outer ring.

To delineate the evolutionary patterns of geographically distinct *L. innocua* strains, we reconstructed a phylogeny based on the conserved amino acid sequences of all single-copy genes (Figure 2). The tree reveals that all strains are monophyletic within the *Listeria* genus, descending from a common ancestor. Notably, despite substantial geographic separation, some strains from different locations clustered within the same clade, indicating close phylogenetic relationships. This suggests that *L. innocua* maintains conserved evolutionary mechanisms and genetic variations in response to environmental stressors, underpinning its high adaptability to diverse niches.

### 3.4. Enrichment Analysis of the Functional Features of Core Genes with GO and KEGG

**Figure 3:**
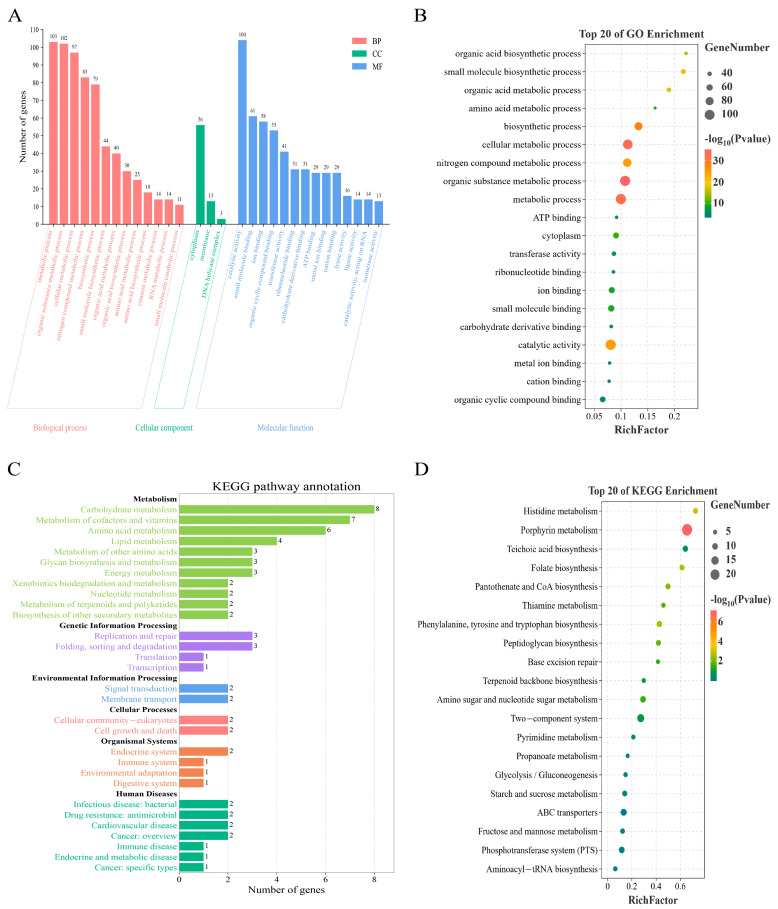
Functional enrichment analysis of core genes in *L. innocua* from distinct geographic regions. (**A**) GO enrichment terms. (**B**) Top 20 significantly enriched GO terms. (**C**) KEGG pathway enrichment. (**D**) Top 20 significantly enriched KEGG pathways.

To explore the functional traits of 2220 core genes in *L. innocua* strains from distinct regions, we employed the GO and KEGG databases for functional annotation and categorization of these genes. The GO database classifies gene functions into 3 primary categories, specifically Biological Processes (BP), Cellular Components (CC), and Molecular Functions (MF). For the BP category, the most enriched biological processes were metabolic process (*n* = 103), organic substance metabolic process (*n* = 102), cellular metabolic process (*n* = 97), and nitrogen compound metabolic process (*n* = 83). In the CC category, cytoplasm (*n* = 56) and membrane (*n* = 13) were the most prevalent cellular components. Within the MF category, catalytic activity (*n* = 104) and small molecule binding (*n* = 61) were the most enriched molecular functions (Figure 3A). A bubble chart was generated to depict the top 20 significantly enriched GO terms from the core gene analysis, ranked by gene count and *p*-value (Figure 3B).

The KEGG pathway database is a public database that is used most widely for metabolic pathway analysis, and it classified 6 categories of biological metabolic pathways: Cellular Processes, Metabolism, Genetic Information Processing, Human Diseases, Environmental Information Processing, and Organismal Systems. The core genes were assigned into 6 categories of KEGG. Among these, the carbohydrate metabolism (*n* = 8), metabolism of cofactors and vitamins (*n* = 7), and amino acid metabolism (*n* = 6) were the most abundant pathways in the Metabolism category. In the Genetic Information Processing category, replication and repair (*n* = 3), and sorting and degradation (*n* = 3) were the most abundant pathways. In the Environmental Information Processing category, signal transduction (*n* = 2) and membrane transport (*n* = 2) were the most abundant pathways. In the Cellular Processes category, cell growth and death (*n* = 2) and cellular community—prokaryotes (*n* = 2) were the most abundant pathways. In the Organismal Systems category, endocrine system (*n* = 2) was the most abundant pathway. In the Human Diseases category, infectious disease: bacterial (*n* = 2), drug resistance: antimicrobial (*n* = 2), cardiovascular disease (*n* = 2), and cancer: overview (*n* = 2) were the most abundant pathways (Figure 3C). A bubble chart was generated to depict the top 20 significantly enriched KEGG pathways from the core gene analysis, ranked by gene count and *p*-value (Figure 3D).

In conclusion, we performed the enrichment analysis of functional features utilizing GO and KEGG databases, and the results indicate that the 2220 core genes are mainly involved in metabolic processes, compound binding and catalytic activity in *L. innocua*. Instances encompass organic substance metabolic process, cellular metabolic process and biosynthetic process. However, functional information of some genes was still unclear, which necessitates further research in the future. The core genes are closely linked to the fundamental life processes and pathogenic potential of *L. innocua* strains, and play a pivotal role in maintaining essential life activities and facilitating infection and invasion.

### 3.5. Distribution of Virulence and Antibiotic Resistance Genes in L. innocua Strains in Distinct Areas

To research the virulence potential of *L. innocua* strains from distinct geographic regions, we predicted genes that encode virulence factors in the entire genome of *L. innocua*. The VFDB forecasting and annotation results revealed that virulence factors of *L. innocua* were assigned to 12 distinct categories, including Toxin, Surface protein anchoring, Regulation, Peptidoglycan modification, Nucleation-promoting factor, Intracellular survival, Immune modulator, Iron uptake, Invasion, Enzyme, Bile resistance, and Adherence.

**Figure 4:**
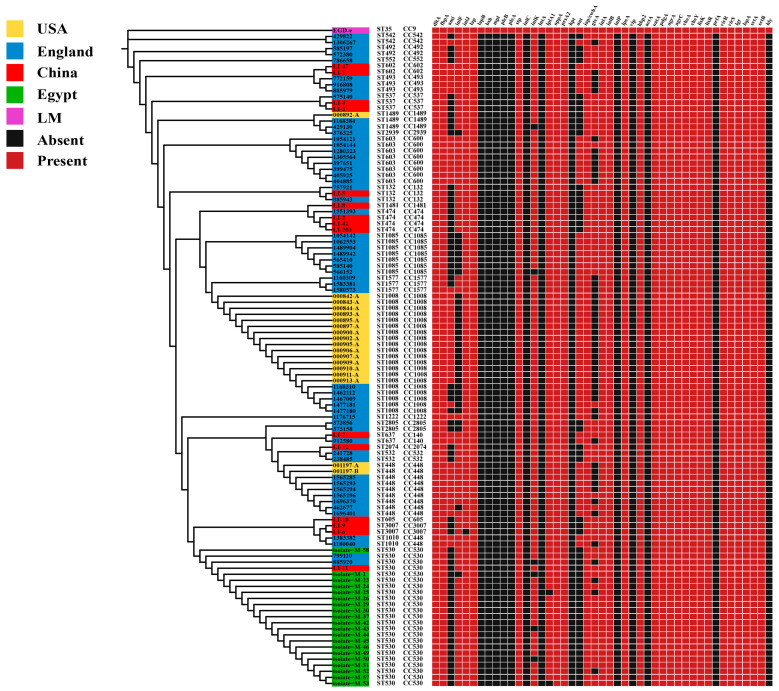
The distribution of virulence genes in *L. innocua* from distinct areas.

In this study, the virulence genes *iap/cwhA*, *inlA*, *inlB*, *lpeA*, *hbp2*, *oatA*, *pdgA*, *agrA*, *agrC*, *cheA*, *cheY*, *lisK*, *lisR*, *virR*, *virS*, *lgt*, *lspA*, *dltA*, *fbpA*, *lap*, *stp*, *oppA*, *prsA2*, *srtA*, and *srtB* were detected in 100% of *L. innocua* strains from distinct areas (Figure 4). Our predictions revealed the presence of numerous key virulence genes, including *inlA* and *inlB*, in *L. innocua* strains from diverse geographic regions. The ubiquitous presence (100%) of these critical genes suggests a significant pathogenic potential. Furthermore, other key virulence factors, such as *inlJ* (99.1%) and *inlK* (94.4%), were also identified at high frequencies. Conversely, the lower prevalence of genes including *hly*, *plcA*, and *plcB* likely accounts for the attenuated virulence of *L. innocua* compared to *L. monocytogenes*. Although *L. innocua* strains are typically considered non-pathogenic, our results indicate that *L. innocua* strains from different regions possess key virulence genes associated with *Listeria* infection. Therefore, *L. innocua* strains may harbor some virulence potential and could potentially exhibit pathogenicity.

**Figure 5:**
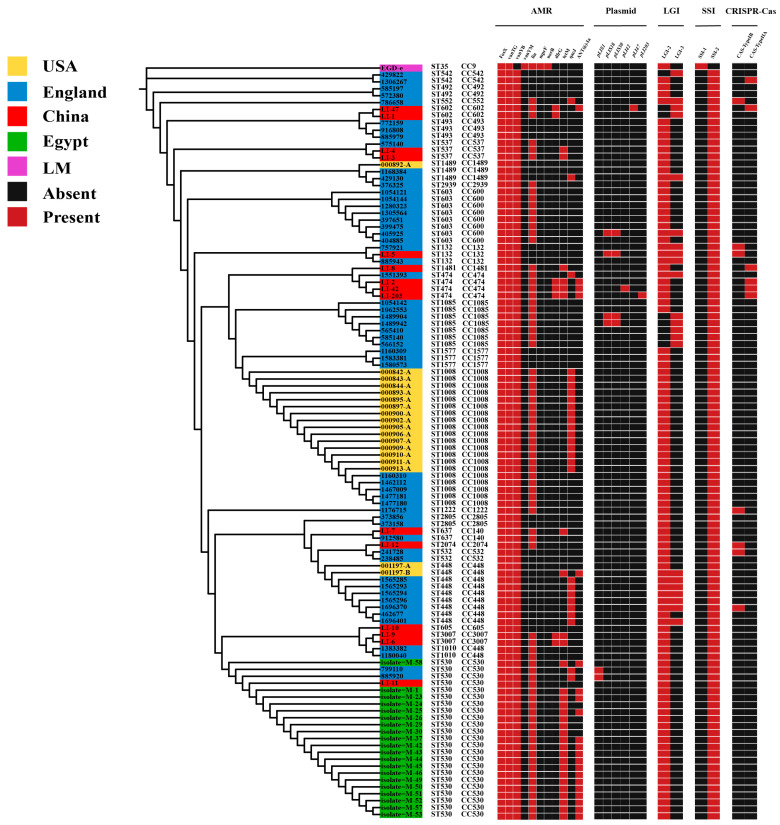
The distribution of antibiotic resistance genes, plasmids, *LGI*, *SSI*, and CRISPR-Cas systems in *L. innocua* from distinct areas.

The extensive use of antibiotics has led to escalating rates of antimicrobial resistance among foodborne bacterial isolates worldwide. Based on the CARD database, we analyzed the distribution of antimicrobial resistance genes in *L. innocua*. In this study, a total of 8 antibiotic resistance genes, which were categorized into 7 drug classes and were involved 5 resistance mechanisms, were identified in the 108 *L. innocua* genomes. Among the *L. innocua* strains from distinct geographic regions, we found that 100% of the strains harbor 2 types of antibiotic resistance genes, namely glycopeptide antibiotic genes (*vanTG*, *vanYB*) and phosphonic acid antibiotic gene (*FosX*) (Figure 5). Compared to *L. monocytogenes*, which contains *vanTG* (100%) and *vanYM* (100%) in glycopeptide antibiotic genes, *L. innocua* strains harbor *vanTG* (100%) and *vanYB* (100%). Additionally, some *L. innocua* strains carry the lincosamide antibiotic gene (*lin*) (72.2%), diaminopyrimidine antibiotic gene (*dfrG*) (6.5%), tetracycline antibiotic gene (*tetM*) (27.8%), disinfectant and antiseptic gene (*qacJ*) (25%), and aminoglycoside antibiotic gene (*ANT(6)-Ia*) (19.4%). However, these resistance genes are not present in all *L. innocua* strains.

### 3.6. Distribution of Plasmids in L. innocua Strains in Distinct Areas

Presently, numerous researchers have started to focus intensely on the vital role of horizontal transfer of MGEs in bacterial genome evolution and acclimatization to particular environmental stressors. For the detection of plasmids, we utilized PLSDB database and only documented the plasmids that had an identity score of 1. In this study, a total of 6 plasmids were identified in the *L. innocua* genome. Plasmid *pLIS1* is found exclusively in ST530, plasmids *pLI42* and *pLI203* are unique to ST474, plasmid *pLI47* is present only in ST602, while plasmids *pLIS18* and *pLIS30* are found in ST603, ST132, and ST1085 (Figure 5). Despite their broad geographic distribution, some *L. innocua* isolates harbor identical plasmids, suggesting a conserved plasmid repertoire across regions.

### 3.7. Distribution of LGIs in L. innocua Strains in Distinct Areas

We predicted *Listeria* genomic islands (*LGI*), identifying *LGI-2* and *LGI-3*. *LGI-2* contains an arsenic resistance gene cassette (*arsR1D2R2A2B1B2*), two additional genes (*arsD1A1*), cadmium resistance gene (*cadA4*), and genes presumably related to DNA integration, binding, and pathogenicity. *LGI-3* integrates the chromosomal cadmium resistance determinants *cadA1C*, flanked by recombinases and Tn3 transposase, along with genes presumed to be involved in DNA integration, binding, transposition, and recombination. The results show that *LGI-2* is present in a high proportion of *L. innocua* strains from various regions (100/108), whereas *LGI-3* is comparatively less frequent (22/108) (Figure 5), indicating a stronger resistance to cadmium and arsenic.

### 3.8. Distribution of SSIs in L. innocua Strains in Distinct Regions

In *Listeria*, the stress survival islands (*SSI*), which are associated with resistance genes, include 2 islands: *SSI-1* and *SSI-2*. The genes *lmo0444*, *lmo0445*, *pva*, *gadD*, and *gadT* belong to *SSI-1*, while the genes *lin0464* and *lin0465* belong to *SSI-2*. The *SSI-1* gene cluster confers tolerance to environmental stressors such as high bile salt concentrations and low pH, while *SSI-2* supports bacterial survival under alkaline and oxidative stress, enhancing persistence in food manufacturing settings. Although initially regarded as restricted to *L. innocua*, *SSI-2* genes have also been identified in ST121-type *L. monocytogenes*. In this study, *SSI-2* was present in *L. innocua* strains from different regions, while *SSI-1* was absent. Conversely, *SSI-1* was present in the *L. monocytogenes* reference strain EGD-e, but *SSI-2* was not (Figure 5).

### 3.9. Distribution of CRISPR-Cas System Types in L. innocua Strains in Distinct Areas

We characterized the CRISPR-Cas systems, known as adaptive immune defenses in bacteria that can also influence virulence, across 108 *L. innocua* genomes. Two distinct system types were identified: CAS-TypeIB (7/108) and CAS-TypeIIA (6/108). A total of nine unique cas genes were identified across these systems, with each type exhibiting a specific gene complement (Table 1) (Figure 5).

### 3.10. Results of the Pathogenicity analysis of L. innocua Strains

The pathogenicity of *L. innocua* strains was assessed using a Galleria mellonella larval model. The Galleria mellonella larval infection model comprised 13 groups (*n* = 10). Twelve groups were challenged with one of the 12 previously described laboratory *L. innocua* strains, respectively, while the control group was injected with PBS. The mortality rates observed after challenge revealed a spectrum of virulence: two strains (16.7%) exhibited 100% mortality, one (8.3%) caused 90% mortality, and one (8.3%) led to 20% mortality. Four strains (33.3%) resulted in 10% mortality, while another four (33.3%) showed no lethality (0% mortality) (Table 2). Statistical analysis by the Chi-square test indicated a highly significant disparity in Galleria mellonella larval mortality (*p <* 0.001), demonstrating that the *L. innocua* strains exhibited substantial variation in their virulence against Galleria mellonella larvae. These results indicate that while the majority of *L. innocua* strains possess limited or negligible pathogenic potential, a subset of strains exhibit significant virulence and pathogenic capacity.

## 4. Discussion

*Listeria* spp., as an important foodborne pathogen, is extensively distributed worldwide and represents a serious hazard to human life and health [29,30]. Although *L. monocytogenes* and *L. ivanovii* are typically considered the pathogenic species among *Listeria* spp., studies have shown that *L. innocua* is commonly found in *Listeria* isolations [2,24]. Furthermore, there is evidence suggesting that *L. innocua* possesses some pathogenic potential [21].

The core and accessory genomes of *L. innocua* were analyzed from whole-genome sequences. The core genome is essential for fundamental life processes and common phenotypic traits, while the accessory genome contributes unique characteristics that, although dispensable for survival, provide critical benefits for niche adaptation and resistance to antibiotics [1,16,31]. Despite the geographic divergence of the *L. innocua* strains, a foundational set of 2220 core genes was conserved, underpinning essential biological functions. Notably, the total core gene number varied by region: 2460 in the USA, 2178 in England, 2475 in China, and 2582 in Egypt. This genomic heterogeneity was further emphasized by the presence of unique, region-specific core genes in each population. These unique genes, likely developed in response to localized environmental pressures, are a primary driver of the distinct phenotypic and adaptive traits observed in strains from different geographic origins [32]. This is also highly intriguing, as it enables exploring differences between *L. innocua* strains in distinct regions, analyzing the unique traits of strains, and further examining the evolutionary patterns of strains in that region to developing targeted regional prevention and control strategies. With the increasing number of genomes, a continuous expansion of the pan-genome is observed alongside a corresponding reduction and eventual stabilization of the core genome. This phenomenon indicates that *L. innocua* carries an open pan-genome, offering a genetic foundation for its adaptability to diverse ecological niches. The core genome, comprising genes universally conserved across all *L. innocua* strains, is hypothesized to be pivotal for fundamental survival and pathogenic processes. These indispensable genes are crucial for viability and invasive infection, underscoring their role in maintaining basic biological functions and virulence. Consequently, investigating the core genome significantly enhances our understanding of the species’ biodiversity and pathogenic potential, offering crucial insights into its evolutionary strategy and survival mechanisms.

To research the biological diversity and evolutionary traits of geographically distinct *L. innocua* strains, we employed MLST analysis. *L. innocua* isolated from food in the USA are primarily of types ST1008 and CC1008, while in England, the predominant types are ST603 and CC600. In China, the main types are ST474 and CC474, and in Egypt, the predominant types are ST530 and CC530. It is evident that the dominant types of *L. innocua* strains vary across different regions. This variation is attributed to the distinct environmental conditions and pressures faced by *L. innocua* strains in each region [5,19]. To adapt to these diverse environmental pressures, *L. innocua* strains undergo evolutionary changes, which result in different dominant types in various regions. Phylogenetic tree construction of *L. innocua* strains from different regions reveals that although the dominant types of *L. innocua* strains vary by region, all strains isolated from food share a common ancestor. Moreover, some strains from different regions fall within the same phylogenetic branch, indicating that despite divergent evolutionary paths due to environmental pressures, there are still notable commonalities among them. This suggests that, despite regional differences, *L. innocua* strains exhibit certain evolutionary similarities.

Typically, *L. monocytogenes* and *L. ivanovii* are regarded as pathogenic strains of *Listeria*, due to their substantial presence of virulence genes [33]. These virulence genes play a critical role in their invasive infection and pathogenic capability. Studies have shown that *L. innocua* strains carry the same virulence genes as those found in pathogenic *L. monocytogenes* strains, suggesting that *L. innocua* may also possess virulence and pathogenic potential [21]. This study identified a significant presence of virulence genes, such as *inlA* and *inlB*, in *L. innocua* strains from different regions, similar to those found in *L. monocytogenes* strains. During *Listeria* infection, *inlA* and *inlB* genes primarily mediate bacterial entry into host cells by binding to E-Cadherin and Met receptors in the gut, leading to cellular internalization and subsequent infection. Our predictions reveal the presence of virulence genes such as *inlA* and *inlB* in *L. innocua* strains from various regions, indicating that these strains possess the potential to invade and cause disease. Although *L. innocua* is typically considered non-pathogenic and harmless, our study demonstrates its latent pathogenic potential. While *L. innocua* is less virulent compared to *L. monocytogenes*, it still possesses a degree of pathogenic potential. This significant finding suggests that, as *L. innocua* strains continue to evolve, their pathogenic potential may increase, potentially leading to their emergence as pathogenic strains. Therefore, in the control and prevention of *Listeria*, it is crucial not only to target *L. monocytogenes* but also to monitor and address *L. innocua*. This discovery enhances our understanding of *L. innocua* and has important implications for future *Listeria* control efforts.

To further explore the biodiversity, virulence, and pathogenic potential of *L. innocua* strains from different regions, we also predicted genes associated with antibiotic resistance and other resistance mechanisms. Prediction results indicate that *L. innocua* strains from different regions exhibit significant resistance to phosphonic acid antibiotics and glycopeptide antibiotics. Therefore, these antibiotics should be avoided in treatment. *L. monocytogenes* strains also show notable resistance to glycopeptide antibiotics; however, unlike *L. innocua*, the resistance genes evolved in *L. monocytogenes* are distinct. In terms of resistance genes, *L. innocua* strains from different regions carry a large number of resistance genes, such as *LGI-2*, *LGI-3*, and *SSI-2*. These resistance genes enhance the ability of *L. innocua* to adapt to various environments, allowing survival in more specialized conditions. Additionally, these genes improve the survival of *L. innocua* during host infection, thereby increasing its potential pathogenicity.

To validate our previous findings on the pathogenic potential of *L. innocua*, an in vivo infection model using Galleria mellonella larvae was employed. The experimental results demonstrated that challenge with *L. innocua* strains resulted in significant larval mortality. This observation confirms that *L. innocua* exhibits pathogenicity in this model organism. This study has certain limitations. First, the analysis relied on *L. innocua* whole-genome sequences obtained from public databases. As these data were generated and submitted by different laboratories worldwide, their quality is inherently heterogeneous; the inclusion of lower-quality assemblies may introduce uncertainties in the genomic comparisons. Second, while the Galleria mellonella larval model provided valuable insights into virulence, future studies should employ mammalian infection models, such as mice, to further corroborate these findings.

This study confirms the pathogenic potential of *L. innocua*, consequently calling for its inclusion in routine food safety surveillance protocols. Future research should focus on elucidating the origins of its virulence genes and conducting extensive epidemiological studies to determine the distribution and prevalence of pathogenic *L. innocua* strains across the food chain, processing environments, and human clinical cases. Collectively, this study sheds light on a previously underappreciated aspect of *L. innocua* biology, addressing a key knowledge gap and underscoring the need for increased awareness regarding its public health implications.

## 5. Conclusions

In summary, we utilized comparative genomic analysis to research the biodiversity, virulence, and pathogenic potential of *L. innocua* from distinct regions. A significant number of virulence genes were identified in *L. innocua* strains, similar to those found in pathogenic *L. monocytogenes* strains. These virulence genes enhance the pathogenicity of *L. innocua*, indicating that *L. innocua* strains from various regions possess both virulence and pathogenic potential. Furthermore, this perspective was confirmed through in vivo experiments, which verified the pathogenic potential of certain *L. innocua* strains. These findings significantly advance our understanding of *L. innocua* by elucidating its virulence and pathogenicity, thereby providing critical insights for public health risk assessments associated with this bacterium.

## Figures and Tables

**Table 1:** Characterization of CRISPR-Cas system types and associated cas genes in *L. innocua* from diverse geographical regions.

Types	Cas Genes	Area	Number	Total
CAS-TypeIB	cas6_TypeI-III, cas8a1b_TypeIB, cas7b_TypeIB, cas5b_TypeIB, cas3_TypeI, cas2_TypeI-II-III	USA	2	7
		England	5	
		China	0	
		Egypt	0	
CAS-type IIA	csn2_TypeIIA, cas2_TypeI-II-III, cas1_TypeII, cas9_TypeII	USA	2	6
		England	1	
		China	3	
		Egypt	0	
Total				13

**Table 2:** Survival of Galleria mellonella larvae after challenge with *L. innocua* strains.

No.	Strain Name	Total Number	Number of Deaths	Number of Survivors	Mortality Rate
1	*LI-1*	10	1	9	10%
2	*LI-2*	10	1	9	10%
3	*LI-3*	10	9	1	90%
4	*LI-4*	10	10	0	100%
5	*LI-5*	10	2	8	20%
6	*LI-6*	10	0	10	0%
7	*LI-7*	10	1	9	10%
8	*LI-8*	10	1	9	10%
9	*LI-9*	10	0	10	0%
10	*LI-10*	10	0	10	0%
11	*LI-11*	10	0	10	0%
12	*LI-12*	10	10	0	100%
13	*PBS*	10	0	10	0%

## Data Availability

The original contributions presented in the study are included in the article/Supplementary Materials; further inquiries can be directed to the corresponding author.

## Supplementary Materials

The following supporting information can be downloaded at: https://www.mdpi.com/article/10.3390/microorganisms13112525/s1, Table S1: Detailed information of *L. innocua* strains from different regions; Table S2: Detailed information on genome size, GC content, number of contigs and N50 of *L. innocua* strains isolated from different regions; Table S3: Detailed information on MLST of *L. innocua* strains isolated from different regions.
