# Supplementary material for: Investigating the Biological Characteristics and Pathogenic Potential of Listeria innocua Isolated from Food Through Comparative Genomics
Source: Microorganisms. 2025 Nov 2;13(11):2525. doi: 10.3390/microorganisms13112525 (PMC12654670; doi:10.3390/microorganisms13112525)
Supplement: Supplementary file 1 [file microorganisms-13-02525-s001.zip › microorganisms-3870518-supplementary.pdf]

Supplementary Material

Investigating the Biological Characteristics and Pathogenic Potential of *Listeria innocua* Isolated from Food Through Comparative Genomics

Bo Zhang [1,2,†], Runlai Cao [1,†], Qilin Wang [1], Pan Hu [2], Yacong Li [1], Ziyu Liu [1], Zhuqing Xue [1], Weiyang Wang [1], Shasha Zhang [3] and Xiaoxu Wang [1,*]

[1] Key Laboratory of Special Animal Epidemic Disease, Ministry of Agriculture and Rural Affairs, Jilin Provincial International Cooperation Key Laboratory for Science and Technology Innovation of Special Animal and Plants, Institute of Special Animal and Plant Sciences, Chinese Academy of Agricultural Sciences, Changchun 130112, China; zbjlu97@163.com (B.Z.); caorunlai@caas.cn (R.C.); wangqilin@caas.cn (Q.W.); lycong910@163.com (Y.L.); 13844790017@163.com (Z.L.); xzqblblbl@163.com (Z.X.); weiyangwang8779@163.com (W.W.)

[2] State Key Laboratory for Diagnosis and Treatment of Severe Zoonotic Infectious Diseases, Key Laboratory for Zoonosis Research of the Ministry of Education, Institute of Zoonosis, and College of Veterinary Medicine, Jilin University, Changchun 130062, China; hupan84@163.com

[3] Panjin Center for inspection and testing, Panjin 124010, China; hbgcyzr@163.com

[*] Correspondence: wangxiaoxussdd@126.com; Tel.: +86-159-4301-5057

[†] These authors contributed equally to this work.

Table S1. Detailed information of *L. innocua* strains from different regions.

Region	Bacterial species	Strain names	Genome size (Mbp)	GC content(%)	No. of contigs	N50(Kbp)	Isolation Source
USA	*Listeria innocua*（n=18）	000842-A 000843-A 000844-A 000892-A 000893-A 000895-A 000897-A 000900-A 000902-A 000905-A 000906-A 000907-A 000909-A 000910-A 000911-A 000913-A 001197-A 001197-B	3.1	37.1	26	523.8	food
England	*Listeria innocua*（n=55）	238485 241728 373158 373856 376325 397651 399475 404885 405925 429130 429822 462677 565410 566152 572380 575140 585140 585197 757921 772159 786658 799110 885920 885943 885979 912580 916808 1054121 1054142 1054144 1062553 1160309 1160310 1168384 1176715 1180040 1280323 1305564 1306267 1383382 1462112 1467009 1477180 1477181 1489904 1489942 1551393 1565285 1565293 1565294 1565296 1580573 1583381 1696370 1696401	3.0	37.4	29	359.1	food
China	*Listeria innocua*（n=15）	LI-1 LI-2 LI-3 LI-4 LI-5 LI-6 LI-7 LI-8 LI-9 LI-10 LI-11 LI-12 LI-42 LI-47 LI-203	2.9	37.4	10	1442.9	food
Egypt	*Listeria innocua*（n=20）	isolate=M-1 isolate=M-23 isolate=M-24 isolate=M-25 isolate=M-26 isolate=M-29 isolate=M-30 isolate=M-37 isolate=M-42 isolate=M-43 isolate=M-44 isolate=M-45 isolate=M-46 isolate=M-49 isolate=M-50 isolate=M-51 isolate=M-52 isolate=M-53 isolate=M-57 isolate=M-58	3.0	37.5	74	131.2	food
	Listeria monocytogenes	EGD-e					Reference genome
Total	109						

Table S2. Detailed information on genome size, GC content, number of contigs and N50 of *L. innocua* strains isolated from different regions.

Region	Strain names	Genome size (Mbp)	GC content(%)	No. of contigs	N50(Kbp)	Strain names	Genome size (Mbp)	GC content(%)	No. of contigs	N50(Kbp)
USA (n=18)	000842-A	3.2	37	25	526.6	000905-A	3.0	37	22	568
	000843-A	3.1	37	34	485.7	000906-A	3.1	37	29	490.8
	000844-A	3.2	37	36	485.7	000907-A	3.0	37.5	21	566.7
	000892-A	3.0	37.5	10	559.4	000909-A	3.0	37	20	567.2
	000893-A	3.1	37	27	512.1	000910-A	3.1	37	28	490.9
	000895-A	3.1	37	30	511.8	000911-A	3.2	37	24	529.1
	000897-A	3.1	37	26	511.8	000913-A	3.1	37	29	490.4
	000900-A	3.1	37	27	486.5	001197-A	3.0	37.5	11	591.7
	000902-A	3.1	37	48	503.3	001197-B	3.1	37.5	13	550.5
England (n=55)	238485	2.8	37.5	64	84.5	1054142	3.0	37.5	17	357.7
	241728	2.9	37.5	22	362.3	1054144	2.9	37.5	19	289.1
	373158	2.9	37.5	18	464.8	1062553	3.0	37.5	17	385
	373856	2.8	37.5	27	286.1	1160309	3.0	37.5	18	288.4
	376325	2.9	37.5	59	143.1	1160310	3.0	37	19	566.6
	397651	3.1	37	30	336	1168384	3.0	37	31	236.4
	399475	3.0	37	20	307.3	1176715	3.0	37.5	22	410.5
	404885	3.0	37	68	83.2	1180040	3.0	37.5	34	204.6
	405925	2.9	37.5	19	393.3	1280323	3.0	37.5	21	306.9
	429130	3.0	37.5	75	94.5	1305564	3.0	37.5	32	179.4
	429822	3.1	37	36	391	1306267	3.3	37.5	25	277.5
	462677	3.1	37.5	50	192	1383382	2.9	37.5	15	370.2
	565410	3.1	37.5	42	462.7	1462112	3.0	37	26	321
	566152	2.9	37.5	119	92.5	1467009	3.0	37	25	340.3
	572380	3.0	37.5	16	398.3	1477180	3.0	37	28	229.3
	575140	2.9	37.5	14	508.5	1477181	3.0	37	20	583.3
	585140	3.0	37.5	47	321.1	1489904	3.0	37.5	14	543.6
	585197	2.9	37.5	14	399.8	1489942	3.0	37.5	16	505.9
	757921	3.0	37.5	20	561.4	1551393	3.2	37.5	27	225.1
	772159	2.9	37.5	45	189	1565285	3.1	37	16	550
	786658	3.1	37.5	21	313.7	1565293	3.1	37	19	550
	799110	3.0	37.5	19	387.5	1565294	3.0	37	21	550
	885920	3.0	37.5	75	125.4	1565296	3.1	37	19	549.8
	885943	3.0	37.5	32	296.3	1580573	2.9	37.5	17	389.4
	885979	3.0	37.5	14	492.1	1583381	3.0	37.5	19	389.4
	912580	3.0	37.5	31	472.6	1696370	3.1	37.5	21	480.9
	916808	3.1	37.5	11	547.2	1696401	3.0	37.5	17	589.7
	1054121	2.9	37.5	18	374.8					
China（n=15）	LI-1	3.0	37.5	18	511	LI-9	2.9	37	15	580.9
	LI-2	2.9	37.5	13	1468.5	LI-10	2.8	37.5	10	536.2
	LI-3	2.8	37.5	9	1480.2	LI-11	2.8	37	9	1474.3
	LI-4	2.8	37.5	9	1480.2	LI-12	2.8	37.5	12	543.5
	LI-5	2.9	37.5	12	1454.6	LI-42	3.0	37.5	2	2900
	LI-6	2.9	37.5	10	1453.2	LI-47	3.1	37.5	3	2900
	LI-7	3.0	37.5	9	1481.2	LI-203	3.0	37.5	2	2900
	LI-8	2.9	37	14	480.4					
Egypt（n=20）	isolate=M-1	3.0	37.5	65	99.5	isolate=M-44	3.1	37.5	25	417.3
	isolate=M-23	3.0	37.5	123	51.7	isolate=M-45	3.0	37.5	58	126.2
	isolate=M-24	2.8	37.5	146	43.6	isolate=M-46	3.0	37.5	34	197.1
	isolate=M-25	3.0	37.5	130	51.3	isolate=M-49	3.0	37.5	84	105.5
	isolate=M-26	2.9	37.5	52	136	isolate=M-50	2.9	37.5	87	83.6
	isolate=M-29	3.0	37.5	43	159.4	isolate=M-51	3.0	37.5	45	131.1
	isolate=M-30	3.0	37.5	20	300	isolate=M-52	2.9	37.5	109	57.8
	isolate=M-37	3.0	37.5	40	188.1	isolate=M-53	3.0	37.5	55	138.1
	isolate=M-42	3.0	37.5	68	84.1	isolate=M-57	3.0	37.5	105	64
	isolate=M-43	3.0	37.5	126	59	isolate=M-58	3.0	37.5	56	131

Table S3. Detailed information on MLST of *L. innocua* strains isolated from different regions.

Region	Strain names	ST	CC	Strain names	ST	CC
USA (n=18)	000842-A	1008	CC1008	000905-A	1008	CC1008
	000843-A	1008	CC1008	000906-A	1008	CC1008
	000844-A	1008	CC1008	000907-A	1008	CC1008
	000892-A	1489	CC1489	000909-A	1008	CC1008
	000893-A	1008	CC1008	000910-A	1008	CC1008
	000895-A	1008	CC1008	000911-A	1008	CC1008
	000897-A	1008	CC1008	000913-A	1008	CC1008
	000900-A	1008	CC1008	001197-A	448	CC448
	000902-A	1008	CC1008	001197-B	448	CC448
England (n=55)	238485	532	CC532	1054142	1085	CC1085
	241728	532	CC532	1054144	603	CC600
	373158	2805	CC2805	1062553	1085	CC1085
	373856	2805	CC2805	1160309	1577	CC1577
	376325	2939	CC2939	1160310	1008	CC1008
	397651	603	CC600	1168384	1489	CC1489
	399475	603	CC600	1176715	1222	CC1222
	404885	603	CC600	1180040	1010	CC448
	405925	603	CC600	1280323	603	CC600
	429130	1489	CC1489	1305564	603	CC600
	429822	542	CC542	1306267	542	CC542
	462677	448	CC448	1383382	1010	CC448
	565410	1085	CC1085	1462112	1008	CC1008
	566152	1085	CC1085	1467009	1008	CC1008
	572380	492	CC492	1477180	1008	CC1008
	575140	537	CC537	1477181	1008	CC1008
	585140	1085	CC1085	1489904	1085	CC1085
	585197	492	CC492	1489942	1085	CC1085
	757921	132	CC132	1551393	474	CC474
	772159	493	CC493	1565285	448	CC448
	786658	552	CC552	1565293	448	CC448
	799110	530	CC530	1565294	448	CC448
	885920	530	CC530	1565296	448	CC448
	885943	132	CC132	1580573	1577	CC1577
	885979	493	CC493	1583381	1577	CC1577
	912580	637	CC140	1696370	448	CC448
	916808	493	CC493	1696401	448	CC448
	1054121	603	CC600			
China （n=15）	LI-1	602	CC602	LI-9	3007	CC3007
	LI-2	474	CC474	LI-10	605	CC605
	LI-3	537	CC537	LI-11	530	CC530
	LI-4	537	CC537	LI-12	2074	CC2074
	LI-5	132	CC132	LI-42	474	CC474
	LI-6	3007	CC3007	LI-47	602	CC602
	LI-7	637	CC140	LI-203	474	CC474
	LI-8	1481	CC1481			
Egypt （n=20）	isolate=M-1	530	CC530	isolate=M-44	530	CC530
	isolate=M-23	530	CC530	isolate=M-45	530	CC530
	isolate=M-24	530	CC530	isolate=M-46	530	CC530
	isolate=M-25	530	CC530	isolate=M-49	530	CC530
	isolate=M-26	530	CC530	isolate=M-50	530	CC530
	isolate=M-29	530	CC530	isolate=M-51	530	CC530
	isolate=M-30	530	CC530	isolate=M-52	530	CC530
	isolate=M-37	530	CC530	isolate=M-53	530	CC530
	isolate=M-42	530	CC530	isolate=M-57	530	CC530
	isolate=M-43	530	CC530	isolate=M-58	530	CC530
